# Cross-cultural adaptation, test–retest reliability, and construct validity of the Thai version of the University of Washington Pain-Related Self-Efficacy Scale

**DOI:** 10.1097/PR9.0000000000000787

**Published:** 2019-12-06

**Authors:** Angkana Khampanthip, Rotsalai Kanlayanaphotporn, Mark P. Jensen, Prawit Janwantanakul

**Affiliations:** a Department of Physical Therapy, Faculty of Allied Health Sciences, Chulalongkorn University, Bangkok, Thailand; b Department of Rehabilitation Medicine, University of Washington, Seattle, WA, USA

**Keywords:** Self-efficacy, Chronic low back pain, Musculoskeletal disorder, Reliability, Validity

## Abstract

**Introduction:**

One psychosocial factor in the biopsychosocial model is pain-related self-efficacy, which has been shown to be a strong predictor of response to pain treatment.

**Objectives:**

To cross-culturally adapt the University of Washington Pain-Related Self-Efficacy Scale (UW-PRSE6) into Thai and evaluate its psychometric properties.

**Methods:**

The study was approved by the Chulalongkorn University Human Ethics Committee (COA No. 156/2018). The original UW-PRSE6 was cross-culturally adapted using the Functional Assessment of Chronic Illness Therapy translation methodology. Two hundred forty-one individuals with chronic low back pain completed the Thai version of UW-PRSE6 (T-UW-PRSE6), Thai Fear Avoidance Beliefs Questionnaire (T-FABQ), and Thai Medical Outcome Study Short-Form 36 (T-SF-36). A subset of 152 participants completed the T-UW-PRSE6 again after a 7-day interval. Cronbach's alpha and intraclass correlation coefficients were calculated to estimate internal consistency and test–retest reliability, respectively. The construct validity of the T-UW-PRSE6 was evaluated by computing Spearman correlation coefficients between the T-UW-PRSE6 score and the measures of the validity criterion variables.

**Results:**

The T-UW-PRSE6 had good internal consistency (Cronbach's alpha = 0.85) and moderate test–retest reliability (intraclass correlation coefficient [2,1] = 0.72). The T-UW-PRSE6 was negatively correlated with the T-FABQ Work and Physical Activity subscales (*r*s = −0.34 and −0.34, respectively) and positively correlated with the General Health, Physical Functioning, Role Physical, Role Emotional, Social Functioning, Bodily Pain, Vitality, and Mental Health scales of the T-SF-36 (*r*s = 0.38, 0.42, 0.54, 0.51, 0.47, 0.54, 0.41, and 0.40, respectively).

**Conclusion:**

The T-UW-PRSE6 demonstrated acceptable psychometric properties for assessing pain-related self-efficacy in individuals with chronic low back pain, making available a measure for facilitating future cross-cultural research on pain self-efficacy.

## 1. Introduction

Chronic pain is a significant public health problem with the annual prevalence in general population ranging from 1% to 51%, depending on the definition and measure of chronic pain used.^5,19,43^ The experience of pain has negative effects on patients' physical and psychological function and often interferes with social relationships.^17^

The biopsychosocial model of chronic pain has been proposed as a framework for understanding the complexity of pain and disability. One of the key psychosocial factors in the model is pain-related self-efficacy, defined as an individual's confidence in their ability to tolerate pain and to participate in daily activities despite pain.^7,8,40^ Measures of pain-related self-efficacy have evidenced stronger associations with chronic pain intensity and disability than indications or measures of physical damage to bodily structures.^8,13,25^ Self-efficacy is also a strong predictor of response to pain treatment.^26,27^ In addition, in longitudinal studies, low self-efficacy has been shown to be a risk factor for poorer subsequent outcomes, including pain exacerbations.^24^ Higher confidence in one's ability to tolerate pain is associated with pain tolerance^30^ and may prevent the development of depressive symptoms in individuals with chronic pain.^14^

(1) A number of measures of self-efficacy have been developed, including the Arthritis Self-Efficacy Scale,^35^ the Chronic Disease Self-Efficacy Scale,^34^ the Chronic Pain Self-Efficacy Scale,^3^ the Pain Self-Efficacy Questionnaire,^40^ and the Self-Efficacy Scale.^1^ Each of these has a number of disadvantages that limit their utility. They were developed using classic measure development theory,^39^ which requires that the measure be administered in full, thus limiting the options for tailored item selection or computer-assisted testing. The unavailability of an item bank (instead of static measures) also makes it challenging to score responses to the items or different combinations of the items into a common metric (usually T-scores, with a mean of 50 and SD of 10 in the normative population), allowing for ease of scale score interpretation and comparisons between different samples, even when or if the specific items from the item bank that were administered from the group differ.^16^
(2) A measure of pain self-efficacy, called the University of Washington Pain-Related Self-Efficacy Scale (UW-PRSE), was recently developed based on more modern scale development approaches (eg, item response theory).^2^ The UW-PRSE items were developed to be easily understood (with a reading level of 4.3th grade) and applicable to individuals with a variety of chronic pain problems, including chronic low back pain (LBP). A 6-item brief version of the UW-PRSE can be used with minimal assessment burden.
(3) For measures of domains that are important to adjustment to chronic pain to be useful in cross-cultural research, it is important that they are translated and culturally adapted to different languages. The aims of this study were to translate the UW-PRSE6 into Thai using the Functional Assessment of Chronic Illness Therapy (FACIT) translation methodology method and to evaluate the test–retest reliability and construct validity of the Thai version of the UW-PRSE6 (T-UW-PRSE6) in patients with chronic LBP. We hypothesized that T-UW-PRSE6 score would be negatively associated with measures of dysfunction (ie, Fear Avoidance Beliefs Questionnaire [FABQ]) and positively associated with measures of adaptive function (ie, Medical Outcome Study Short-Form 36 [SF-36]).

## 2. Method

### 2.1. Participants and procedures

A convenience sample of individuals with chronic LBP from hospitals in the Bangkok metropolitan area was recruited to evaluate the psychometric properties of a number of measures that were translated into Thai, including the PRSE6 which is the focus of the current study. The study inclusion criteria were as follows: aged 18 years or older, being a native Thai speaker who can read and speak the Thai language, and having chronic LBP, as defined by the NIH Task Force on Research Standards for Chronic Low Back Pain as “a back pain problem that has persisted at least 3 months and has resulted in pain on at least half the days in the past 6 months.”^15^ Exclusion criteria included having a serious medical condition or complication in addition to LBP that might affect the ability to participate in the study procedures. All potential participants were screened through interview using a standardized form to collect their demographic and pain history information (ie, age, sex, height, weight, pain location, duration of pain, diagnoses, and employment status).

To assess the reliability and construct validity of the T-UW-PRSE6, each participant was asked to complete the T-UW-PRSE6 items twice with at least a 7-day interval in between. They were asked to complete an 11-point Global Perception of Change (GPC) scale^29^ at the second assessment. Respondents rated the overall change in their condition since the last assessment on a −5 to 5 scale, with −5 = “vastly worse” and 5 = “completely recovered.” For purposes of evaluating the test–retest reliability of the T-UW-PRSE6, only the subset of the sample who reported little or no change in their condition over the 1-week interval, ie, scoring from −1 to 1 on the GPC scale, was included. The study was approved by the Chulalongkorn University Human Ethics Committee (COA No. 156/2018).

### 2.2. University of Washington Pain-Related Self-Efficacy Scale

As indicated previously, pain-related self-efficacy has been defined as a person's confidence in his or her ability to manage pain and minimize the impact of pain on various aspects of their lives, including physical and psychological functioning (eg, sleep, fatigue, and mood), activities (eg, leisure activities and self-care), and participation (eg, work responsibilities, social interactions, and relationships).^2^ The UW-PRSE6 items assess the respondent's perceived ability to (1) perform daily activities despite pain, (2) manage pain, (3) engage in valued activities despite pain, (4) keep pain from interfering with their social life, (5) stay in a good mood despite pain, and (6) get a good night's sleep despite pain.^2^ Respondents indicate their agreement with each self-efficacy item on a 5-point Likert scale with 1 = “not at all,” 2 = “a little bit,” 3 = “somewhat,” 4 = “quite a bit,” and 5 = “very much.” The total raw score when all 6 items are administered can range from 6 to 30, with higher scores indicating higher levels of pain-related self-efficacy. However, the scores from the items administered—whether 1 item is administered or more than 1 item is administered—are normally transformed to a T-score, with a mean of 50 and SD of 10 in the normative sample (made up of individuals with varying chronic pain conditions).^2^

### 2.3. Cross-cultural translation

**Figure 1.**
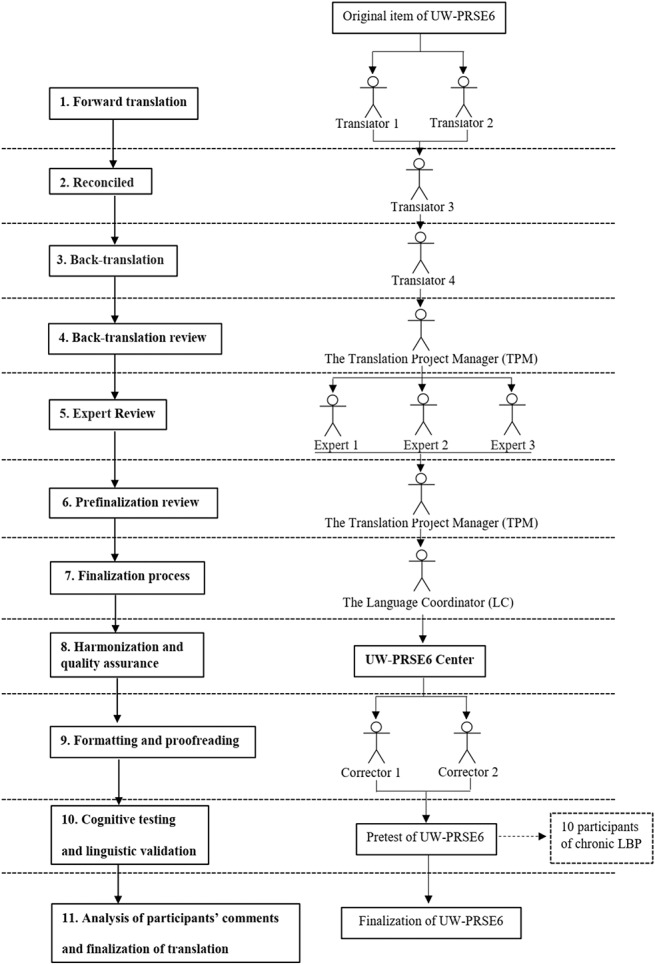
Modified FACIT methodology diagram. LBP, low back pain.

The translation team implemented specific steps to develop precise and culturally appropriate translations based on the English source. The UW-PRSE6 items were translated into Thai following the FACIT translation methodology.^18^ The FACIT translation methodology consists of 11 steps^52^ (Fig. 1).

(1) Forward translation: Source items in English were translated into Thai by 2 bilingual native Thai speakers. The translators were asked to use simple language appropriate to Thai culture. The translators completed each item themselves to get a better understanding of the meaning and interpretation of the items.
(2) Reconciliation: A third native Thai speaker reconciled any differences in the translations between the first 2 translated questionnaires. After that, the most suitable translation was generated, and the reasons for supporting the reconciliation were recorded by this individual.
(3) Back-translation: The reconciled Thai version was back-translated by a native English-speaking translator who was also fluent in Thai. The back translator was blind to the original source English version. The translator performed the translation using simple language that captured the literal meaning of the items.
(4) Back-translation review: A native English speaker who had experience in the development of the UW-PRSE6 performed a review of the back-translation. The source and the back-translated English versions were compared to assess the equivalence of the English source and Thai translation. The Translation Project Manager (TPM), who is a health professional with experience in the intent of the items and a native Thai speaker, provided comments on the discrepancies which helped clarify the intent behind the items.
(5) Expert reviews: Three bilingual Thai native speakers who are health care professionals examined all of the preceding steps and selected the most appropriate translation for each item or developed alternate translations if none of the previous translations were deemed acceptable.
(6) Prefinalization review: The TPM evaluated the merit of the reviewers' comments. Any potential problems in their recommended translations were identified and commented upon to guide the Thai Language Coordinator (LC).
(7) Finalization process: The LC, who is a health professional with experience in the intent of the items and a native Thai speaker, determined the final translation by reviewing the preceding information as well as the TPM's comments. The LC provided explanations for the choice of final translation. In addition, the respective literal back-translation and polished back-translation for each item were provided.
(8) Harmonization and quality assurance: A native English speaker who was involved in the development of the UW-PRSE6 made a preliminary assessment of the accuracy and equivalence of the final translation and verified that the documentation of the decision-making process was complete. The LC was consulted again for additional input when necessary.
(9) Formatting and proofreading: Formatting, typesetting, and proofreading of all items of the final translation were checked for spelling and grammatical issues or item forms by 2 proofreaders who worked independently and reconciled the proofreading comments.
(10) Cognitive testing and linguistic validation: The final version of the Thai version of the UW-PRSE6 was pretested. The objective was to verify that the meaning of each item was equivalent to the English source after translation. An interview script in Thai was created by the TPM. Ten Thai native speakers with chronic pain participated in this step. Each participant completed the questionnaire independently. They were then asked to provide feedback on the difficulty and appropriateness of each item. Participants were asked questions regarding item comprehension (ie, the meanings of specific words in the items, the overall meaning of the item, or why they chose a specific answer). When appropriate, the participants were asked to provide alternative wording for any items that they indicated were difficult to understand.
(11) Analysis of participants' comments and finalization of translation: The TPM compiled participants' comments (back-translated into English) and summarized the issues. All the participants' comments and suggestions were examined and considered by the TPM to determine whether the items were well understood by Thai participants. In consultation with the LC, final revisions were made to the translation. Finally, the native English speaker who was involved in the development of the UW-PRSE6 conducted a final quality review, and the translation was finalized.

### 2.4. Test–retest reliability

We evaluated test–retest reliability by computing test–retest correlation coefficients for the T-UW-PRSE6 scores among individuals with chronic LBP, who indicated on an 11-point GPC scale that their condition did not change between 2 assessments made at least 7 days apart.

### 2.5. Internal consistency

Cronbach's alpha was computed to estimate the homogeneity of all the items.

### 2.6. Construct validity

For evaluating convergent validity, a priori hypotheses were formulated regarding associations between the T-UW-PRSE6 scores and measures of domains thought to be similar and related to the construct of pain-related self-efficacy. One of the measures used to evaluate the construct validity of the T-UW-PRSE6 was the Thai version of the 16-item Fear Avoidance Beliefs Questionnaire (T-FABQ), which was designed to measure fear-related beliefs regarding the effects of physical activity on the experience of pain in individuals with LBP.^41^ With the FABQ, respondents indicate their level of agreement with each item reflecting fear of movement on a 7-point Likert scale, with 0 = “completely disagree” and 6 = “completely agree.” The FABQ has 2 scales, assessing fear of movement related to work (Work scale) and fear of movement associated with physical activity (Physical Activity scale). The FABQ Work scale can range from 0 to 42, and the FABQ Physical Activity scale ranges from 0 to 24. A higher score indicates more strongly held fear avoidance beliefs.^47,48,50^ If the T-UW-PRSE6 scale was valid, we anticipated negative moderate to strong associations between it and the 2 FABQ scales. The FABQ was administered at the initial assessment only.

The Thai version of Medical Outcome Study Short-Form 36 (T-SF-36) is a self-administered questionnaire containing 36 items that assess 8 quality of life domains. The scale score labels are General Health, Physical Functioning, Role Limitations Related to Physical Problems, Role Limitations Related to Emotional Problems, Social Functioning, Bodily Pain, Vitality, and Mental Health.^11,28^ Items assessing each domain are coded, summed, and transformed on to a scale from 0 to 100, with higher scores indicating better quality of life or health status for that domain. The 2-week test–retest reliabilities of the T-SF-36 have been shown to be comparable with those of the original scale, ranging from 0.60 to 0.81.^28^ If the T-UW-PRSE6 scale was valid, we anticipated positive and statistically significant associations between it and all of the T-SF-36 scales. The T-SF-36 was administered at the initial assessment only.

### 2.7. Statistical analyses

We first computed the mean values and SDs (for continuous variables) and numbers and rates (for categorical variables) for the demographic and pain history variables to describe the sample. We then computed the Cronbach's alpha for the T-UW-PRSE6 items to estimate its internal consistency. We computed intraclass correlation coefficients to estimate the test–retest stability of the T-UW-PRSE6 in the subsample who reported little to no change in their condition over the 1-week interval.^46^ Construct validity of T-UW-PRSE6 was evaluated by computing Spearman correlation coefficients between the T-UW-PRSE6 scale score and the measures of the validity criterion variables, including the 2 subscales of FABQ scores and the 8 subscales of SF-36 scores. All the statistical analyses were conducted using SPSS statistical software, version 22.0 (SPSS, Inc, Chicago, IL). Statistical significance was set at the 5% level.

## 3. Results

Two hundred forty-one participants with chronic LBP enrolled in the study. The descriptive characteristics of participants are presented in Table 1. As can be seen, the majority of the sample was women (71%), and the sample had an average age of 46.2 (SD = 16.9) years. The majority of participants (80%) were employed. Their average LBP duration was 52.3 (SD = 76.4) months.

**Table 1:**
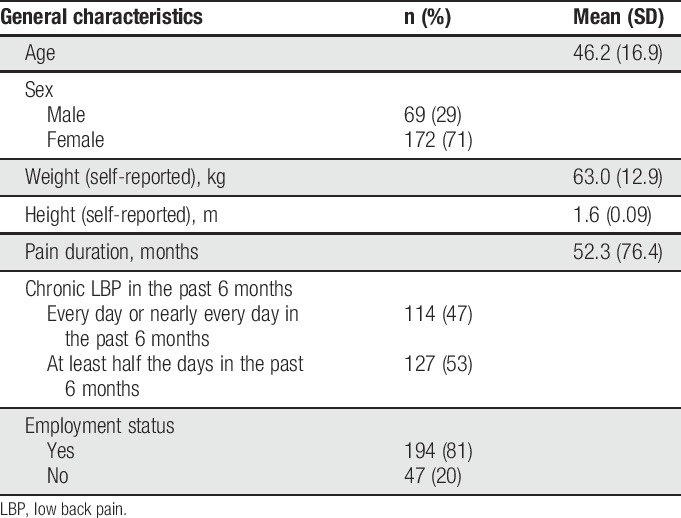
Characteristics of participants (n = 241).

### 3.1. Internal consistency

The Cronbach's alpha of the T-UW-PRSE6 was 0.85, indicating a good internal consistency for the measure in the study sample.^4^

### 3.2. Test–retest reliability

One hundred fifty-two of the participants rated their overall condition as having little to no change at the second assessment. The mean T-UW-PRSE6 score at the baseline and second assessments for these individuals was 53.2 (±7.4) and 53.5 (±7.0), respectively (*P* = 0.507). The overall test–retest reliability (intraclass correlation coefficient [2,1]) of the T-UW-PRSE6 in this subsample was 0.72, indicating moderate to good test–retest reliability.^44^

### 3.3. Construct validity

Two hundred forty-one participants with chronic LBP completed all measurement instruments at the initial assessment. Spearman correlations between the T-UW-PRSE6 and the measures of the validity criterion variables are presented in Table 2. As can be seen, for convergent validity, significant negative correlations were found between the T-UW-PRSE6 score and the FABQ Work (*r* = −0.34) and Physical Activity (*r* = −0.34) scales (all *P*s < 0.01). A significant positive correlation was found between the T-UW-PRSE6 scores and the T-SF-36 General Health (*r* = 0.38), Physical Functioning (*r* = 0.42), Role Physical (*r* = 0.54), Role Emotional (*r* = 0.51), Social Functioning (*r* = 0.47), Bodily Pain (*r* = 0.54), Vitality (*r* = 0.41), and Mental Health (*r* = 0.40) scales (all *P*s < 0.01).

**Table 2:**
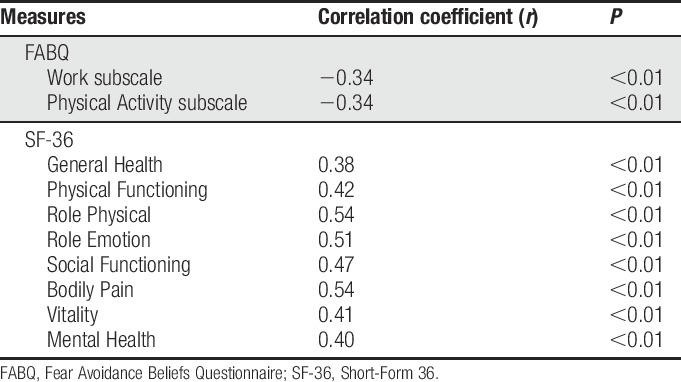
Spearman correlation coefficient between the T-UW-PRSE6 and the validity criteria measures (n = 241).

## 4. Discussion

The aims of this study were to translate the UW-PRSE6 into Thai using the FACIT methods for cross-cultural adaptation and evaluate the psychometric properties of the Thai version of UW-PRSE6 in individuals with chronic LBP. The findings support the successful cross-cultural adaptation process of the UW-PRSE6 instructions and items into Thai. In addition, the findings showed that the T-UW-PRSE6 has good internal consistency, moderate to good test–retest reliability, and construct validity, as evidenced by significant associations with measures of the validity criterion variables, including the 2 FABQ subscale scores and the 8 T-SF-36 subscale scores.

We used the FACIT translation methodology to ensure that we complied with the highest quality translation and adaptation procedures.^20,23,52^ One aspect of the FACIT translation methodology that makes it unique among translation procedures is its emphasis on a universal translation approach whenever possible, which addresses the challenge of dealing with languages that are spoken in multiple countries. The FACIT translation methodology also provides opportunities for significant dialogue between the reviewer/language coordinator and the project manager, during which item translations are discussed and explored, ensuring appropriate decision making regarding the translation and cross-cultural adaptation of each item.^45^

The process of translation and back-translation of the Thai version of UW-PRSE6 was performed strictly in accordance with the established guidelines.^9,36^ Backward translation was used to correct possible misinterpretations of forward translation and confirm conceptual equivalence. In the review process, we compared the translated instructions and items in all steps with the original version and found that the meaning of some items from the back-translation process had different meanings from the original version. Consequently, the items were revised to ensure that they communicated the meaning of the original version. For example, “Deal with the pain you have during your everyday activity” was changed to “Manage the pain you have during your everyday activity” because the literal meaning of “deal with” in Thai does not communicate the same meaning as in English.

The results indicated that, as hypothesized, the T-UW-PRSE6 is conceptually related to the T-FABQ and T-SF-36 subscales. The study findings indicating significant negative associations between the T-UW-PRSE6 scores and the FABQ subscale scores are consistent with those from a previous study show a significant negative association between a measure of pain self-efficacy and fear avoidance in an English-speaking sample of patients with chronic LBP.^6^ Participants with higher self-efficacy have also been found to have less fear of movement, catastrophizing, avoidance of pain, less pain severity, disability, and depression.^24,38,42,51^

Our findings indicating significant and positive associations between the T-UW-PRSE6 score and the SF-36 subscale scores are consistent with those from studies using English-speaking samples, which also found positive associations between measures of pain-related self-efficacy and measures of different quality of life domains, such as activities of daily living, mobility, and physical function.^10,31,32^ Consistent with previous studies, the findings also support the importance of pain-related self-efficacy as a predictor of adjustment to chronic pain. Higher pain-related self-efficacy levels are consistently associated with better scores in quality of life, high activity levels, reduced disability, lower pain intensity, less pain behaviors, and worked longer hours.^24,33,38,40,49^

Low back pain is an extremely important health problem in the general population. One-year prevalence rates for LBP range between 17% and 33%, whereas estimates for lifetime prevalence range from 11% to as high as 84%.^22^ Despite substantial investment in improving workplace ergonomics and other preventive measures over the past few decades, the 1-year prevalence of chronic LBP has been reported to range from 15% to 45%, with a point prevalence of 30%.^37^ Moreover, LBP is a leading cause of disability globally, causing personal suffering and impaired quality of life and work in general. Thus, LBP constitutes a major socioeconomic burden on both patients and society. The impact of LBP is more pronounced in low-income and middle-income countries, including Thailand, where the population is increasing and aging rapidly and adequate resources to address the problem might be limited.^12^ Measures of pain-related self-efficacy—a key psychosocial factor in biopsychosocial models of chronic pain—are essential as outcome variables to facilitate scientific knowledge for addressing the severity and impact of chronic pain in Thailand and other developing countries.

The T-UW-PRSE6 is a health status measure designed to be completed by patients to assess self-efficacy due to chronic pain. The T-UW-PRSE6 is brief, easy to complete, and readily understood by patients. The results of the current study indicate that the T-UW-PRSE6 can be translated and culturally adapted into the Thai language without significant modification of the contents and questionnaire structure. All participants in the current study could complete the questionnaire by themselves, demonstrating its ease and comprehensibility in our sample of Thai individuals with chronic LBP.

However, there are a number of study limitations that should also be considered when interpreting the study findings. First, the T-UW-PRSE6 was tested in a sample of patients with chronic LBP. Thus, we were unable to evaluate its reliability and validity in individuals with other types of chronic pain problems. Although we do not have any reasons to anticipate that it would evidence significant different levels of reliability and validity, say, in individuals with chronic headaches or with other chronic pain conditions, research to evaluate its psychometric properties in these populations is needed to establish the generalizability of the current findings. Second, we did not evaluate the measure's ability to detect changes in pain-related self-efficacy after treatments designed to target this construct. Sensitivity to change is an important psychometric feature, especially when the measure assesses a domain that is a target of treatment and/or is hypothesized to be a mediator of effective treatment.^21^ Future research to evaluate the sensitivity of the T-UW-PRSE6 to change in a variety of samples with chronic conditions would be useful and would provide important additional information regarding the measure's utility. Third, we were not able to identify any cutoffs for determining the T-UW-PRSE6 scores needed for having positive effects of self-efficacy on pain intensity and disability; knowing such cutoffs would have clinical utility, providing clinicians with information that would be useful for identifying patients who might benefit most from interventions designed to increase self-efficacy (ie, patients who score below the identified cutoff scores). Future researchers should therefore seek to identify T-UW-PRSE6 cutoff scores that can be used for this purpose.

## 5. Summary

Despite the study's limitations, the findings provide important initial support for the cultural appropriateness and psychometric properties of the T-UW-PRSE6 scale as a measure of pain-related self-efficacy in individuals with chronic LBP who speak Thai. Additional research would be useful that replicates the current findings in samples of individuals with different chronic pain conditions, that evaluates the sensitivity of the T-UW-PRSE6 to treatment which is designed to change pain-related self-efficacy beliefs, and that identifies cutoffs that would be useful for identifying patients with chronic pain who might most benefit from treatment. Despite this, the measure seems to have enough strength to make it useful for cross-cultural research evaluating the role that pain-related self-efficacy may play and can be used in both clinical treatment and research settings for evaluating pain-related self-efficacy in adjustment to chronic pain.

## Disclosures

The authors have no conflict of interest to declare.

This study was funded by the 90th Anniversary of Chulalongkorn University Fund (Ratchadaphiseksomphot Endowment Fund).
